# Prognostic factors in Polish patients with *BRCA1*-dependent ovarian cancer

**DOI:** 10.1186/s13053-015-0041-2

**Published:** 2016-01-23

**Authors:** Wiktor Szatkowski, Paweł Blecharz, Jerzy W. Mituś, Marek Jasiówka, Elżbieta Łuczyńska, Jerzy Jakubowicz, Tomasz Byrski

**Affiliations:** 1 Department of Gynaecological Oncology, Centre of Oncology, Maria Skłodowska-Curie Memorial Institute, Kraków Branch, ul. Garncarska 11, 31-115 Kraków, Poland; 2 Department of Surgical Oncology, Centre of Oncology, Maria Skłodowska-Curie Memorial Institute, Kraków Branch, ul. Garncarska 11, 31-115 Kraków, Poland; 3 Department of Anatomy, Collegium Medicum, Jagiellonian University, ul. Kopernika 12, 31-034 Kraków, Poland; 4 Department of Medical Oncology, Centre of Oncology, Maria Skłodowska-Curie Memorial Institute, Kraków Branch, ul. Garncarska 11, 31-115 Kraków, Poland; 5 Department of Radiology, Centre of Oncology, Maria Skłodowska-Curie Memorial Institute, Kraków Branch, ul. Garncarska 11, 31-115 Kraków, Poland; 6 Department of Radiotherapy, Centre of Oncology, Maria Skłodowska-Curie Memorial Institute, Kraków Branch, ul. Garncarska 11, 31-115 Kraków, Poland; 7 Department of Genetics and Pathology, International Hereditary Cancer Center and Clinic of Oncology Pomeranian Medical University, Szczecin, ul. Połabska 4, 70-115 Szczecin, Poland

**Keywords:** Ovarian cancer, *BRCA1*, Prognostic factors

## Abstract

**Background:**

Treatment outcomes appear to be better for ovarian cancer (OC) patients carrying the *BRCA1/2* germline mutation than for patients with sporadic OC. However, most published data are for North American, British and Jewish populations. There have been very few studies on treatment outcomes in Central and Eastern European patients with OC. The aim of this study was to analyse prognostic factors in Polish patients with *BRCA1*-dependent OC (*BRCA1*-OC).

**Methods:**

The records of patients with OC treated with surgery and chemotherapy at the Centre of Oncology in Kraków, Poland, between 2004 and 2009 were reviewed. Based on family history, a group of 249 consecutive patients fulfilling the criteria for risk of hereditary OC were selected and tested for the germline *BRCA1* mutation. Response to combination therapy (surgery and chemotherapy) in the *BRCA1*-OC group was assessed based on clinical examination, imaging and serum CA125.

**Results:**

Germline *BRCA1* mutations were detected in 69 of the 249 patients, but three of these patients failed to complete the study. Finally, 66 patients with *BRCA1*-OC were included in the study group. The median age of the study patients was 49.5 years. All had undergone primary or interval cytoreductive surgery and chemotherapy. Progression occurred in 48 (72.7 %) of the 66 patients and median time to progression was 20 months. The 5-year overall survival rate in was 43.9 % and median survival time was 32.3 months. On multivariate analysis, the endometrial subtype of OC and serum CA125 < 12.5 U/ml at the end of treatment were independent, positive prognostic factors for 5-year overall survival.

**Conclusion:**

Prognostic factors for favourable treatment outcomes in Polish patients with *BRCA1*-OC do not appear to differ from those in patients with sporadic OC. The incidence of the endometrial subtype of OC was relatively high (34.9 %) among women in the study. This was unexpected and has not been reported previously. This subtype of OC was an independent prognostic factor for favourable treatment outcomes.

## Background

Many reports suggest that the outcomes of treatment for ovarian cancer (OC) differ between patients who are carriers of *BRCA1/2* mutations and patients with sporadic OC (SOC). Most investigators report a better outcome for patients with *BRCA1/2*-dependent OC (*BRCA1/2*-OC). The *BRCA1* and *BRCA2* mutations are thought to cause an impaired ability to repair DNA damage, and as a result, *BRCA1/2*-OC is more sensitive than SOC to chemotherapeutic drugs that act directly on the DNA double helix, such as cisplatin and carboplatin [1, 2].

To date, most studies of the effect of *BRCA1* and *BRCA2* mutations on OC treatment outcomes have been carried out among American, British and Ashkenazi Jewish women. The significance of germline mutation of the *BRCA2* gene in the Polish population has not yet been established and, unlike mutation of the *BRCA1* gene, it is not routinely screened for among OC patients. Apart from one observational study in a small group of patients, there are no data on treatment outcomes for Central and Eastern European women with *BRCA1*-OC [3]. It is possible that genetic diversity might lead to different treatment outcomes in this population compared with previously studied populations. Moreover, there are limited data available on prognostic factors among patients with *BRCA1*-OC and it has not yet been determined whether prognostic factors in patients with *BRCA1*-OC differ from those in patients with SOC. Therefore, the aim of this study was to analyse the prognostic factors for favourable treatment outcomes in Polish patients with *BRCA1*-OC.

## Methods

The records of all patients with OC treated with surgery and chemotherapy at the Centre of Oncology in Kraków, Poland, from 2004 to 2009 were reviewed. In total, there were records for 1225 patients with OC diagnosed based on examination of the surgical specimen or biopsy. From the 1225 records, a group of 249 consecutive patients who fulfilled the criteria of high risk for hereditary OC based on family history were identified. After written, informed consent was obtained blood was collected from these 249 patients for *BRCA1* mutation analysis. Exons 2, 5, 11 and 20 of the *BRCA1* gene were analysed, and the most frequent *BRCA1* gene mutations among the Polish population were searched for: 4153delA, 5382insC C61G, 185delAG and 3819del5. The DNA analysis was carried out using denaturing high-performance liquid chromatography (dHPLC), restriction fragment length polymorphism (RFLP) and sequencing. The patients with *BRCA1* mutations were selected as the study group. The study was approved by the Ethics Committee of Maria Skłodowska-Curie Memorial Institute.

Response to combination therapy (surgery and chemotherapy) used to treat *BRCA1*-OC was assessed based on clinical examination, imaging (Response Evaluation Criteria in Solid Tumors [RECIST] criteria 1.0) and marker analysis (serum CA125 concentration). Patients were evaluated every three months for 2 years and every 6 months thereafter. All patients in the study group were observed for at least 3 years or until death.

The effectiveness of treatment was evaluated according to time to progression, defined as the period between the beginning of treatment and clinical or imaging identification of recurrence of cancer, and 5-year overall survival from the beginning of treatment. The mean follow-up period was 65 months. Survival probability was estimated using the Kaplan–Meier method [4]. Peto’s log-rank test was used to assess the statistical significance of differences among the results [5]. The level of statistical significance was set at *p* < 0.05. The Cox proportional hazards model was used to assess the impact of selected factors on patient survival [6].

## Results and discussion

Among the 249 consecutive patients at high risk of hereditary OC who were screened for germline mutation of the *BRCA1* gene, 69 (27.7 %) had mutations. However, three women from this group did not complete the treatment and were excluded from the study (one refused chemotherapy and two died shortly after surgery without having completed adjuvant treatment). Therefore, the final study group included 66 patients with *BRCA1*-OC. Before beginning treatment, all patients had a histopathological diagnosis of OC. The median age was 49.5 years (mean 48 years, range 23–75 years). All study patients underwent planned combination therapy and were further observed. They received from three to nine courses of adjuvant therapy. Population, microscopic and clinical characteristics of the 66 patients with *BRCA1*-OC are presented in Table 1.

**Table 1:** Population, microscopic and clinical characteristics of *BRCA1*-OC patients

	No. of patients	Percent
Population, microscopic and clinical characteristics	66	100
Age		
- ≤ 50 years	34	51.5
- > 50 years	32	48.5
Menopause		
- yes	28	42.4
- no	38	57.6
No. of births		
- 0	10	15.2
- 1	27	40.9
- ≥2	29	43.9
Family history		
- 1st degree relatives with ovarian cancer	8	12.1
- 1st degree relatives with breast cancer	17	25.8
Coexisting breast cancer		
- yes	9	13.6
Type of *BRCA1* gene mutation		
-C61G	31	47
- 5382insC	21	31.8
- 4153delAG	6	9.1
- 189delAG	4	6.1
- 3819del5	2	3
- IVS20 + 60ins12	1	1.5
- 4158A > G	1	1.5
Grading of the tumor		
- G1	2	2.7
- G2	18	27.3
- G3	46	70
Histological type		
- serous	27	40.9
- endometrial	23	34.9
- undifferentiated	8	12.1
- mucous	4	6.1
- clear cell	2	3
- mezonefroid	2	3
Staging FIGO (2009)		
- I	4	6.1
- II	11	16.7
- III	50	75.7
- IV	1	1.5
Cytoreduction		
- primary	44	66.7
- interval	22	33.3
The overall extent of cytoreduction		
- optimal	34	51.5
- suboptimal	32	48.5
The average concentration of CA125 at the beginning of treatment	1113 U/ml	
The average concentration of CA125 at the end of treatment	71 U/ml	

**Fig. 1:**
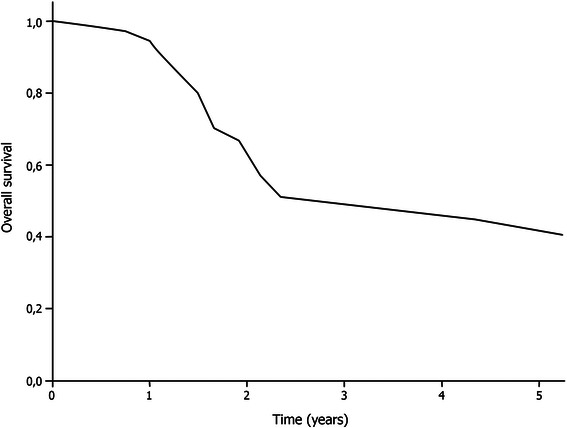
Overall survival of patients with BRCA1-OC

During follow-up, progression occurred in 48 (72.7 %) of the study group. The mean time to progression was 26.2 months (median 20 months). The 5-year survival rate was 43.9 % and the median survival time was 32.3 months. The overall survival Kaplan–Meier curve is presented in Fig. 1. Table 2 shows a comparison of the most significant publications on *BRCA1/2-*dependant OC in terms of survival.

**Table 2:** Comparison of long-term outcome in patients with *BRCA-*OC

Author, year of publication	No. of patients	Population	Overall 5-year survival	Median survival
Rubin 1996 [23]	53	*BRCA1/2*-OC	~60 %^a^	~80 months^a^
Pharoah 1999 [7]	127	*BRCA1/2*-OC	21 %	20,6 months
Boyd 2000 [10]	67	*BRCA1*-OC	~50 %^a^	~60 months^a^
Ramus 2001 [24]	15	*BRCA1*-OC	~25 %^a^	52 months
Cass 2003 [11]	33	*BRCA1/2*-OC	65 %	91 months
Majdak 2005^b^ [3]	18	*BRCA1*-OC	~33 %^a^	~28 months^a^
Chetrit 2008 [8]	213	*BRCA1/2*-OC	46 %	53,7 months
Tan 2008 [25]	22	*BRCA1/2*-OC	~65 %^a^	8,4 years
Kringen 2005 [26]	30	*BRCA1/2*-OC	33,3 %	-
Lacour 2011 [27]	95	*BRCA1/2*-OC	~25 %^a^	101,7 months
Hyman 2012 [9]	30	*BRCA1*-OC	~60 %^a^	6 years
Vencken 2013 [28]	245	*BRCA1*-OC	62 %	6 years
Bolton 2012 [29]	909	*BRCA1*-OC	44 %	4,6 years
Own material	66	*BRCA1*-OC	43,9 %	32,3 months

^a^ Data extrapolated from the Kaplan-Meier curves

^b^ Polish patients population

The population, microscopic and clinical factors that were significant on univariate analysis are presented in Table 3. Multivariate analysis showed a positive, statistically significant impact of the endometrial subtype of OC and serum CA125 (<12.5 U/ml at the end of treatment) on 5-year overall survival from *BRCA1-*OC. Both of these factors were independent prognostic factors for 5-year overall survival (Table 4).

**Table 3:** Results of treatment of patients with *BRCA1*-OC depending on the population, microscopic and clinical characteristics

Population, microscopic and clinical characteristics	No. of patients	5-year overall survival	
		No. of patients	%
The number of patients with *BRCA1* gene mutation	66	29	43.9
^a^ Coexisting neoplasms			
- no	57	28	49.1
- breast cancer	9	1	11.1
^a^ The total extent of cytoreduction			
- optimal	34	20	58.8
- suboptimal	32	9	28.1
^a^ Staging FIGO (2009)			
- I	4	4	100.0
- II	8	5	62.5
- III	50	19	38.0
- IV	4	1	25.0
^a^ Histological type			
- serous	27	11	40.7
- endometrial	23	14	60.9
- undifferentiated	8	1	12.5
- mucous	4	2	50.0
- clear cell	2	1	50.0
- mezonefroid	2	0	0
^a^ CA125 concentration at the beginning of treatment			
- < 285,5	31	21	67.7
- ≥285,5	35	8	22.9
^a^ CA125 concentration at the end of treatment			
- <12,5	34	22	64.7
- ≥12,5	22	7	31.8

^a^ Statistically significant differences, log rank test, *p* <0.05

**Table 4:** Results of the multivariate analysis of prognostic factors in the group of 66 patients with *BRCA1*-OC

Variable	Variant	Relative risk (RR)	Confidence interval	*p* value
Histological type	Undifferentiated adenocarcinoma	39,29	2,07–743,35	*p* = 0.014
	Serous adenocarcinoma	1		
Histological type	Endometrial adenocarcinoma	0,2	0,04–0,85	*p* = 0.03
	Serous adenocarcinoma	1		
Level of CA125 at the end of treatment	<12,5 U/ml	0,23	0,07–0,69	*p* = 0.01

The composition of the study group did not differ from previously published studies in terms of population, histopathologic and clinical factors. The number of *BRCA1-*OC patients in the present study was relatively high compared to previous studies, most of which included 13–43 patients, with only a few recruiting higher numbers of 88–245 patients. Among these previous studies, a small number evaluated the clinical features and outcome of patients with *BRCA1*-OC separately from patients with *BRCA2*-OC. However, these studies were conducted among Jewish, American and Western European populations, which seem to be genetically different from the Polish population [7–9].

Consistent with previous reports, the serous subtype of OC was predominant among the study patients (*n* = 27, 40.9 %). The percentage of the serous subtype reported in the literature varies widely from 25 to 93 %, which might be related to the size of patient groups. However, in contrast to previous reports, the percentage of patients with the endometrial subtype of OC (34.9 %) was high in the present study, whereas previous studies have found that 14 % or less of patients with *BRCA1*-OC have this subtype [10]. Interestingly, another Polish population analysis of patients with *BRCA1*-OC reported an unusually high percentage of mucinous cancers (17 %). However, that study was small with only 18 patients [3]. Both findings are unusual because somatic tumour mutations of the *BRCA1* gene are rare in endometrial and mucinous subtypes of OC.

### Prognostic factors in patients with *BRCA1*-OC

To date, few studies have investigated prognostic factors associated with *BRCA1/2*-OC. Most research has focussed on differences in treatment outcomes between OC patients with and without *BRCA1/2* mutations. Furthermore, few studies have looked at differences in prognostic factors between patients with SOC and *BRCA1/2*-OC.

The strongest prognostic factor for treatment outcomes in patients with SOC is the FIGO cancer stage. On univariate analysis in the present study, the FIGO cancer stage was a prognostic factor for 5-year overall survival of *BRCA1*-OC patients. However, this was not confirmed in the multivariate analysis, possibly as a result of the small number of patients with early-stage disease (4 patients had stage I disease and 8 had stage II disease).

Similar to SOC patients, in the present study optimal surgical cytoreduction (remnants after surgery ≤1 cm) had a positive prognostic impact on survival in patients with *BRCA1*-OC. The difference in the 5-year survival rate between patients with and without optimal surgical cytoreduction was 30 %, which is similar to that reported in the literature for SOC [11, 12]. Only one previous study investigated the impact of the extent of surgical cytoreduction on survival in patients with *BRCA1/2*-OC [10]. That study identified a statistically significant higher risk of death among patients with surgery that was not considered complete, with a hazard ratio (HR) = 1.48 on multivariate analysis. However, in the present study, the prognostic value of cytoreduction was not confirmed on multivariate analysis, probably because of the limited number of cases.

Of the patients with *BRCA1*-OC in the present study, nine (13.6 %) patients had been previously treated for breast cancer, and had achieved a complete and long-lasting remission. In all nine patients, the breast cancer was treated with surgery and one line of anthracycline-based chemotherapy. None of the patients had received platinum agents. Imaging, clinical findings, serum markers and immunohistochemical analysis of ovarian tumours confirmed that all nine patients had primary OC rather than secondary breast cancer. The 5-year survival rate in this group was significantly lower compared with the other *BRCA1*-OC patients (11.1 % vs. 49.1 %, respectively). It should be noted that progression of OC can mask the recurrence of breast cancer resulting in poorer outcomes.

Among patients with *BRCA1*-OC, Chetrit et al. found that the serous subtype of OC was associated with a poorer prognosis compared to the non-serous subtype (5-year overall survival rate of 44.9 % vs. 50 %, respectively), and poorly differentiated tumours were associated with a poorer prognosis than well differentiated and moderately differentiated subtypes (5-year overall survival rate of 45.4 % vs. 55 %, respectively) [8]. In terms of the importance of microscopic subtypes of OC on prognosis, the results of the present study agreed with previous studies on SOC. On univariate analysis the endometrial subtype of OC was associated with a better prognosis compared to the serous subtype of OC, and the poorest prognosis was associated with the undifferentiated adenocarcinoma subtype. The numbers of patients in the present study with mucous, clear-cell and mesonephroid types of *BRCA1*-OC were too small to draw statistical conclusions. Endometrial tumours are more likely to develop in the pelvis without spread to the upper abdomen and they are more likely to be diagnosed at an early stage. Similarly, on multivariate analysis, a statistically significant positive impact on the 5-year overall survival was associated with the endometrial subtype of the OC. The impact of histological grade (G) on treatment outcomes was not statistically significant on univariate or multivariate analysis.

The serum CA125, both at the beginning of treatment and after treatment completion, is a widely confirmed prognostic factor in patients with OC [13–20]. For patients with *BRCA1*-OC, the reported average serum CA125 before treatment ranges from 445 U/ml to 824 U/ml [11, 21, 22]. However, there are no data on serum CA125 levels at the end of treatment. In the present study, the average serum CA125 in patients with *BRCA1*-OC was 113 U/ml and 71 U/ml before and after treatment, respectively. No previous studies have reported that serum CA125 levels were a prognostic factor in patients with *BRCA1/2*-OC. However, on univariate analysis in the present study a relatively low serum CA125 at the beginning of treatment and after treatment was associated with better prognosis (67.7 % vs. 22.9 % 5-year overall survival for serum CA125 ≤ 285.5 U/ml vs. >285.5 U/ml at the beginning of treatment, and 64.7 % vs. 31.8 % 5-year overall survival for serum CA125 of ≤12.5 U/ml vs. >12.5 U/ml after treatment).

## Conclusions

Prognostic factors for treatment outcomes in Polish patients with *BRCA1*-OC do not appear to differ from those in patients with SOC. The incidence of the endometrial subtype of OC was relatively high, which is a new finding not previously reported in the literature. This form of OC was also an independent, positive prognostic factor on multivariate analysis.
